# Echocardiography-Derived Hemodynamic Forces Are Associated with Clinical Outcomes in Patients with Non-Ischemic Dilated Cardiomyopathy

**DOI:** 10.3390/jcm13133862

**Published:** 2024-06-30

**Authors:** Marco Cesareo, Eduard Ródenas-Alesina, Andrea Guala, Jordi Lozano-Torres, Guillem Casas, Fabrizio Vallelonga, Lorenzo Airale, Ignacio Ferreira-González, Alberto Milan, Jose F. Rodriguez-Palomares

**Affiliations:** 1 Hypertension Unit, Division of Internal Medicine, University Hospital Città della Salute e della Scienza of Turin, Via Genova 3, 10126 Turin, Italy; lorenzo.airale@gmail.com; 2 Department of Medical Sciences, University of Turin, Via Verdi 8, 10124 Turin, Italy; vallelonga.fabrizio@gmail.com (F.V.); alberto.milan@unito.it (A.M.); 3 Cardiology Department, Vall d’Hebron University Hospital, Passeig de la Vall d’Hebron 119-129, 08035 Barcelona, Spain; eduard.rodenas@vallhebron.cat (E.R.-A.); jordilozano87@gmail.com (J.L.-T.); guillem.casas@vallhebron.cat (G.C.); iferregon@gmail.com (I.F.-G.); jfrodriguezpalomares@gmail.com (J.F.R.-P.); 4 Department of Medicine, Universitat Autonoma de Barcelona, Passeig de la Vall d’Hebron 119-129, 08035 Barcelona, Spain; 5 Centro de Investigación Biomédica en Red-Enfermedades Cardiovaculares (CIBERCV), Av. de Monforte de Lemos, 3-5, 28029 Madrid, Spain; andrea.guala@vhir.org; 6 Vall d’Hebrón Research Institute (VHIR), Pg. de la Vall d’Hebron, 119-129, 08035 Barcelona, Spain; 7 Division of Internal Medicine, Candiolo Cancer Institute-Fondazione del Piemonte per l’Oncologia (FPO)-Istituto di Ricovero e Cura a Carattere Scientifico (IRCCS), Strada Provinciale 142, KM 3.95, 10060 Turin, Italy; 8 Consorcio de Investigación Biomédica en Red de Epidemiología y Salud Pública (CIBERESP), Av. Monforte de Lemos, 3-5, 28029 Madrid, Spain

**Keywords:** hemodynamic forces, intraventricular pressure gradients, echocardiography, dilated cardiomyopathy, speckle tracking

## Abstract

**Introduction**: Non-ischemic dilated cardiomyopathy (NIDCM) is characterized by a reduced left ventricular (LV) ejection fraction (LVEF, <50%) and a high risk for heart failure (HF) and death. Echocardiography-derived hemodynamic forces (HDFs) may provide important information on LV mechanics, but their prognostic value is unknown. **Aim**: To explore the features of echocardiography-derived HDFs in NIDCM and their association with clinical endpoints. **Methods**: Asymptomatic, non-hospitalized NIDCM patients free from coronary artery disease and moderate or severe valvular heart disease were included in this single-center observational retrospective longitudinal study. Those with atrial fibrillation and a follow-up <12 months were excluded. Major adverse cardiovascular events (MACE) were defined as a composite of all-cause death, HF hospitalization, and ambulatory intravenous diuretics administration. LV HDFs were analyzed with a prototype software. Apex-base (HDFs-ab), lateral-septal (HDFs-ls), and HDFs-angle were computed. **Results**: Ninety-seven patients were included, sixty-seven (69%) were males, mean age was 62 ± 14 years, and mean LVEF was 39.2 ± 8.6%. During a median follow-up of 4.2 (3.1–5.1) years, 19 (20%) patients experienced MACE. These patients had a higher HDFs-angle (71.0 (67.0–75.0) vs. 68.0 (63.0–71.0)°, *p* = 0.005), lower HDFs-ls (1.36 (1.01–1.85) vs. 1.66 ([1.28–2.04])%, *p* = 0.015), but similar HDFs-ab (5.02 (4.39–6.34) vs. 5.66 (4.53–6.78)%, *p* = 0.375) compared to those without MACE. in a Cox regression analysis, HDFs-angle (HR 1.16 (95%-CI 1.04–1.30), *p* = 0.007) was associated with MACE, while other conventional echocardiography parameters, including LVEF and LV longitudinal strain, were not. **Conclusions**: HDFs-angle is associated with clinical endpoints in NIDCM. A higher HDFs-angle may be a marker of impaired myocardial performance in patients with reduced LVEF.

## 1. Introduction

Non-ischemic dilated cardiomyopathy (NIDCM) represents a group of myocardial disorders characterized by reduced left ventricular (LV) systolic function, mainly measured by left ventricular ejection fraction (LVEF), in the absence of coronary artery disease, hypertension, or valvular heart disease [1,2], bearing a high risk for heart failure (HF) and death [3,4]. Transthoracic echocardiography (TTE) is essential for diagnosis, follow-up, and prognosis assessment in these patients. Some of the previously described TTE variables associated with HF admission and mortality in NIDCM are LVEF, LV global longitudinal strain (GLS), and right ventricular dilatation and dysfunction [1,5,6]. GLS is also associated with prognosis and undiagnosed CAD [7]. However, none of these parameters consider the hemodynamic features of the LV.

**Figure 1:**
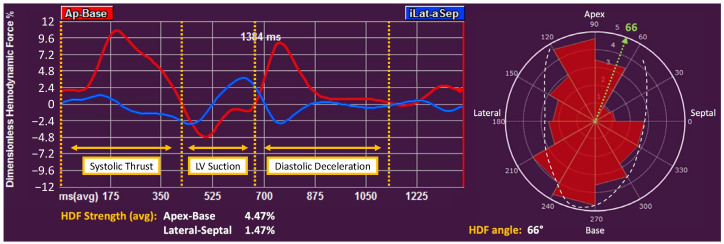
Example of hemodynamic force (HDFs) analysis with Medis QStrain software. On the left: waveforms of apex-base HDFs (red) and lateral-septal HDFs (blue) during the cardiac cycle. The three main time intervals of apex-base HDFs are highlighted in yellow. On the right: polar diagram of HDFs according to their orientation and strength (red triangles; the larger the triangle the stronger the HDFs). The green dotted line represents the average vectorial HDFs orientation (angle) calculated throughout the whole cardiac cycle. Ap-Base: Apex-base oriented HDFs; iLat-aSep: lateral-septal oriented HDFs.

Recent studies have introduced the evaluation of the LV hemodynamic forces (HDFs) as a promising method for the mechanistic, non-invasive assessment of the interaction between the LV and intra-cardiac blood motion [8,9], which could be of value in the study of various cardiomyopathies [9,10,11,12]. The average intraventricular pressure gradients inside the LV cavity can be estimated using a mathematical model that, exploiting the balance of momentum, integrates LV geometry and wall motion with the areas of aortic and mitral valve orifices [8,13]. Projected into specific directions in the LV, these HDFs can be decomposed into longitudinal (i.e., apex-base, HDFs-ab) and transversal (i.e., lateral-septal, HDFs-ls) components. The relative magnitude of longitudinal and transversal forces can be assessed in terms of an angle (HDFs-angle), a vectorial combination of these components that represents the global orientation of HDFs, ranging from 0° (totally transversal) to 90° (totally longitudinal) [8,9] (Figure 1). Alterations in HDF magnitude or orientation were reported in dilated and other cardiomyopathies [11,14,15], and more recently their impairment as assessed by cardiac magnetic resonance (CMR) demonstrated prognostic potential in NIDCM patients [16].

The aim of this study was to explore the features of TTE-derived HDFs in NIDCM and test their potential prognostic value in NIDCM.

## 2. Materials and Methods

This is a single-center, observational, retrospective, longitudinal cohort study including consecutive patients diagnosed with NIDCM that underwent a TTE between January 2015 and December 2019 at Vall d’Hebron University Hospital (Barcelona, Spain). Inclusion criteria were LV ejection fraction (LVEF) <50%, absence of coronary artery disease as assessed by invasive coronary angiography and CMR when clinically indicated, and no evidence of congenital heart disease or greater than mild aortic or mitral valvular disease according to the latest guidelines [17]. In order to avoid the possible confounding effect of fluid overload and irregular heartbeat on HDFs analysis or prognosis, patients were excluded if during the first available TTE they were symptomatic for HF (New York Heart Association class > II), had atrial fibrillation, or if patients were lost to follow-up before 12 months. The last clinical contact for the present study occurred on 31 October 2022.

The study complies with the Declaration of Helsinki. The study protocol was reviewed and approved by the local Institutional Ethics Committee (PR(AG)379/2022, date of approval 16 January 2023).

### 2.1. Clinical Endpoint

All patients underwent a clinical assessment with ECG and TTE evaluation at the Cardiomyopathies Unit of the Cardiology Department and were clinically followed-up. The follow-up time started at the time of the index TTE and ended at the last medical contact. The main endpoint of the study was a composite of major adverse cardiovascular events (MACE) that included all-cause mortality, HF hospitalization, or the need for intravenous diuretics. HF hospitalization was defined as a hospitalization with a diagnosis of HF according to the treating physicians. Full medical history and medications were collected at index TTE, and Charlson comorbidity index was calculated [18].

### 2.2. Echocardiography

TTE images were acquired with commercially available ultrasound machines (e.g., Vivid E95, GE Healthcare, Milwaukee, WI, USA) and reviewed by specialists in cardiac imaging and cardiomyopathies. Conventional TTE parameters were assessed according to the current guidelines [19]. LV mass and LV relative wall thickness (RWT) were estimated according to LV end-diastolic diameter, and the thicknesses of the interventricular septum and infero-lateral wall. LV end-diastolic and end-systolic volumes, LVEF, and left atrial volume were measured using Simpson’s biplane technique from apical two- and four-chamber views. Sphericity index was calculated as the ratio of mean LV diastolic mid-cavity diameter to mean LV diastolic length measured on apical two-, three-, and four-chamber views. Dubois and Dubois’ formula was used to calculate body surface area (BSA). LV mass (LVMi), LV end-diastolic volume (LV EDVi) and left atrial volume (LAVi) were indexed for BSA. LV diastolic function parameters LAVi, mitral inflow peak velocities of early (E) and late (A) diastolic filling on pulsed-wave Doppler, and tissue-Doppler imaging of the septal and lateral mitral annulus (E′) were measured according to the current recommendations [20].

### 2.3. Deformation Imaging and Hemodynamic Forces

LV HDFs as well as global longitudinal (GLS) and circumferential (GCS) LV strains were analyzed on apical two-, three-, and four-chamber views. Left atrial (LA) strain and right ventricle free wall strain were calculated according to an apical four-chamber view. All speckle-tracking measurements were performed using a dedicated software (QStrain Echo, Medis Medical Imaging, Leiden, The Netherlands). The mathematical model used for HDFs calculation has been described in detail elsewhere [8]. LV geometry and endocardial velocity information were derived from LV speckle-tracking analysis. Briefly, endocardial borders are traced semi-automatically after identifying the LV base and apex in conventional apical two-, three-, and four-chamber views, while aortic and mitral valve orifices areas are derived from the internal diameters of the annulus of the valves, which are measured using a parasternal long-axis view. Speckle-tracking technology is used by the software to determine endocardial border velocity, while the average blood velocity across the valves is derived from volumetric changes in the LV and the valves orifice areas, enforcing the conservation of mass principle. After this process, a frame-by-frame intraventricular pressure gradient is projected into apex-base and lateral-septal HDF waveforms. Average HDFs-ab, HDFs-ls, and HDFs-angle over the whole cardiac cycle are calculated. HDFs-ab is further assessed in three time intervals (Systolic Thrust, LV Suction, and Diastolic Deceleration) (see Figure 1) [8,9], while HDFs-angle is also calculated in the first two. The positive reversal of the negative HDFs-ab LV Suction wave is also recorded [16]. Strain and HDFs measurements are performed by a MC who is blinded to patients’ outcomes at the time of the analysis.

### 2.4. Statistical Analysis

The normal distribution of variables was tested using the Kolmogorov–Smirnov test. Data were presented as mean ± standard deviation or median [interquartile range], or as observations (percentage frequency) where appropriate for continuous and categorical variables, respectively. Differences between groups were analyzed by t-test for continuous normally distributed data or Mann–Whitney test for continuous non-normally distributed data and the Yates’ χ^2^ test or Fisher exact test were used to test for categorical variables. Baseline demographic, clinical, and imaging data were compared according to the occurrence of MACE. Correlations between variables were assessed using the Pearson’s correlation coefficient for continuous normally distributed data or the Spearman’s rank correlation coefficient for continuous non-normally distributed data. Survival analysis to estimate MACE-free survival were obtained using the Kaplan–Meier method and compared using the log-rank test. Patients were followed until the occurrence of MACE or until their last follow-up visit, at which point they were censored. Time to event analysis was performed by using the Cox proportional hazard regression method. Hazard ratios (HR) with corresponding 95% confidence intervals (CI) were reported in the univariate analysis. To avoid overfitting, bivariate analyses adjusting separately for potential confounders were performed. Variables with *p* ≤ 0.200 on the univariate Cox regression analysis were considered as potential confounders. Changes in the −2Log Likelihood method were used to determine the incremental predictive value of HDFs over other variables. The optimal thresholds of the HDF parameters were assessed according to their sensitivity and specificity for MACE using the Youden index. A *p* < 0.05 for two-tailed tests was considered statistically significant. Statistical analysis was performed during November 2022 by using a dedicated software (IBM SPSS, Version 25.0, Armonk, NY, USA).

## 3. Results

**Figure 2:**
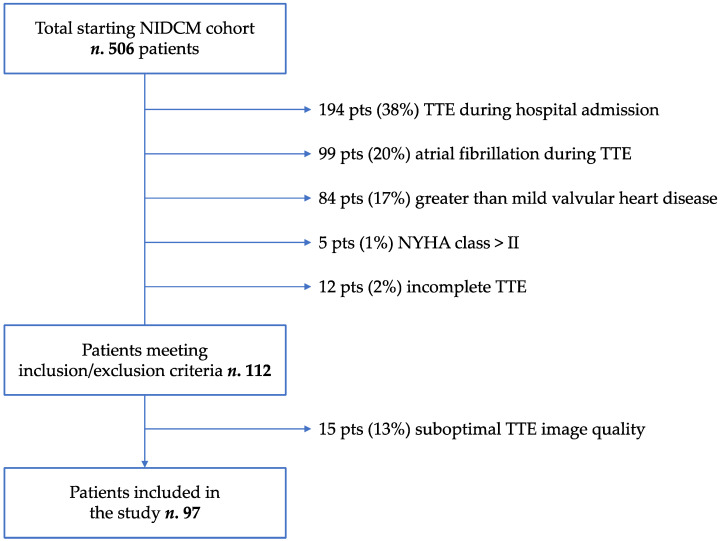
Patients’ inclusion flow chart. NIDCM: non-ischemic dilated cardiomyopathy; TTE: transthoracic echocardiography.

The initial cohort consisted of 506 consecutive NIDCM patients. Out of a total of 112 patients who complied with the inclusion and exclusion criteria, 15 (13%) were excluded for suboptimal TTE image quality; therefore, 97 patients were finally included in the study (Figure 2). Sixty-seven (69%) patients were male, mean age was 62 ± 14 years, and twenty-eight (29%) had diabetes. Mean LVEF was 39.2 ± 8.6%, and mean GLS was −13.6 ± 3.6%.

During a median follow-up of 4.2 (3.1–5.1) years, 19 (20%) patients experienced at least one MACE, of which there were 3 deaths, 12 HF hospitalizations, and 4 cases of intravenous diuretic administration.

Clinical and echocardiographic characteristics of the study population are shown in Table 1. Compared to patients who did not experienced MACE, those with MACE were significantly older, had lower BSA, and had a higher prevalence of diabetes, hypertension, and wide QRS. Conventional echocardiographic parameters were comparable between the two groups, except for the E/e’ ratio and TAPSE. However, none of the subgroups presented with a right ventricular dysfunction.

Data on HDFs and their comparisons between the two groups are summarized in Table 1. Median HDFs-ab, HFD-ls, and HDFs-angle were 5.58 (4.4–6.7)%, 1.60 (1.3–2.0)%, and 68.0 (63.5–71.0)°, respectively. There was no difference in HDFs-ab between patients who experienced a MACE (5.0 (4.4–6.3)%) compared to those who did not (5.7 (4.5–6.8)%, *p* = 0.375). Conversely, patients with MACE had significantly lower HDFs-ls (1.4 (1.0–1.9) vs. 1.7 (1.3–2.0)%, *p* = 0.015), and higher HDFs-angle (71.0 (67.0–75.0) vs. 68.0 (63.0–71.0)°, *p* = 0.005). Furthermore, HDFs-angle during Systolic Thrust and LV Suction were higher in patients with MACE, while no differences were observed in the HDFs-ab sub-phases. These results suggest that the globally transversal component of intraventricular pressure gradient is lower in patients with MACE, with similar longitudinal components, resulting in a higher HDFs-angle, a possible subclinical marker of impaired myocardial mechanics.

On further analysis, considering patients with LVEF ≥ 35% (n. 70), both HDFs-ab and HDFs-ls were lower in patients who experienced MACE (n. 14, 20%) than in those who did not (5.2 (4.6–6.5) vs. 6.0 (5.5–7.6)%, *p* = 0.111, and 1.22 (0.93–1.89) vs. 1.68 (1.28–2.04)%, *p* = 0.015, respectively), while HDFs-angle was higher in patients with MACE (74 (69–75) vs. 69 (65–71), *p* = 0.008).

ACE: angiotensin-converting enzyme; ARBs: angiotensin II-receptor blockers; BMI: body mass index; BP: blood pressure; BSA: body surface area; CKD: chronic kidney disease; COPD: chronic obstructive pulmonary disease; E wave: early filling wave at mitral inflow Doppler imaging; E/A ratio: ratio between E wave and atrial filling wave at mitral Doppler imaging; E/e′ ratio: ratio between E wave and average septal and lateral early wave at tissue Doppler imaging (e’); GCS: global circumferential strain; GLS: global longitudinal strain; HDFs: hemodynamic forces; LA: left atrium; LAVi: LA volume indexed for BSA; LV: left ventricle; LV EDVi: LV end-diastolic volume indexed for BSA; LVEF: LV ejection fraction; LVMi: left ventricular mass indexed for BSA; MRAs: mineralocorticoid receptor antagonists; TAPSE: tricuspid annular plane systolic excursion; RV FWS: right ventricle free wall strain; RWT: relative wall thickness.

### 3.1. Correlation between Hemodynamic Forces and Clinical and Echocardiographic Variables

The relationship between HDFs and conventional TTE parameters was investigated (see Tables S1 and S2, Figure S1). Both HDFs-ab and HDFs-ls showed significant correlation with age (R = −0.302, *p* = 0.003 and R = −0.290, *p* = 0.004, respectively) and QRS duration (R = −0.201, *p* = 0.049 and R = −0.203, *p* = 0.047, respectively), while HDFs-angle was not correlated with any clinical variable. HDFs-ab was significantly correlated with LVEF (R = 0.629, *p* < 0.001), GLS (R = −0.577, *p* < 0.001), GCS (R = −0.609, *p* < 0.001), sphericity index (R = −0.283, *p* = 0.006), LVMi (R = −0.204, *p* = 0.045), RWT (R = 0.223, *p* = 0.028), and LA reservoir strain (R = 0.208, *p* = 0.040). Similarly, HDFs-angle demonstrated significant correlation with LVEF (R = 0.438, *p* < 0.001), GLS (R = −0.460, *p* < 0.001), GCS (R = −0.405, *p* < 0.001), sphericity index (R = −0.383, *p* < 0.001), and with LV EDVi (R = −0.279, *p* = 0.006) and LAVi (R = −0.232, *p* = 0.022). HDFs-ls showed no significant correlation with any echocardiographic parameter. HDFs-angle correlated with both HDFs-ab and HDFs-ls (R = 0.360, *p* < 0.001 and R = −0.488, *p* < 0.001).

### 3.2. Clinical Endpoints

Univariate Cox proportional hazard ratios are summarized in Table 2. In the univariate analysis, age, male sex, BSA, hypertension, diabetes, Charlson comorbidity index, hemoglobin, and the use of loop diuretics were significantly associated with higher risk of MACE. Among conventional TTE parameters RWT, TAPSE, and E/e’ ratio were significantly associated with the endpoint.

HDFs-ls (HR 0.27 (0.09–0.80), *p* = 0.019) and HDFs-angle (HR 1.16 (1.04–1.30), *p* = 0.007) were significantly associated with MACE, while HDFs-ab was not (0.86 (0.66–1.13), *p* = 0.290). HDFs-angle during LV Suction was associated with MACE (HR 1.05 (1.00–1.11), *p* = 0.041), while other HDFs sub-phases were not. Given these results and considering that the HDFs-angle of the whole cardiac cycle may summarize the relative importance of HDFs-ab and HDFs-ls and their sub-phases, it was selected as the main parameter for the subsequent analyses.

In the bivariate Cox regression analyses the HDFs-angle remained significantly associated with MACE after individual adjustment for all variables potentially associated (*p* ≤ 0.200) with the endpoint in the univariate analysis (Table 2 and Table S3 and Figure S2). Specifically, the HDFs-angle showed an incremental predictive value over the variables more strongly associated with the endpoint, such as age, Charlson comorbidity index, hemoglobin, and conventional TTE parameters, such as LVEF, LV EDVi, GLS, TAPSE, LA reservoir strain, and E/E’ ratio (all *p* < 0.05 for change in −2Log Likelihood, Table 3).

**Figure 3:**
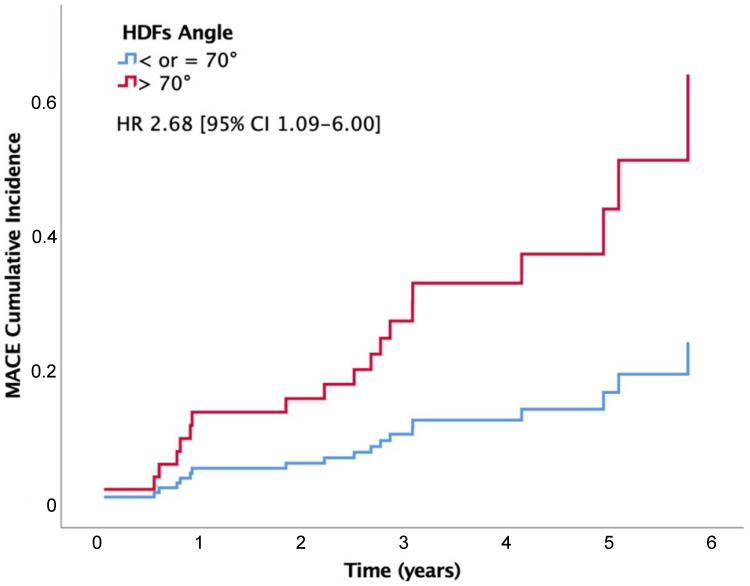
Cox logistic regression curves for cumulative incidence of major adverse cardiovascular events (MACE) in patients with hemodynamic forces angle (HDFs-angle) > 70° (red) and ≤ 70° (blue).

According to the Youden index, the optimal threshold to discriminate MACE by HDFs-angle in this specific population was 70°: an HDFs-angle higher than 70° was present in 10 patients (52.6%) with MACE and in 20 (25.6%) without MACE (*p* = 0.022). A value ≤ 70° provided 74% specificity and 87% negative predictive value. An HDFs-angle higher than 70° was significantly associated with increased risk of MACE (HR 2.68 (95% CI 1.09–6.60)) (Figure 3). Mean event-free survival time was 6.11 vs. 4.95 years for patients with an HDFs-angle ≤ and > 70°, respectively (Log rank *p* = 0.026). At a median follow-up time of 4.2 years, event-free survival was 88% in patients with an HDFs-angle ≤ 70° and 66% in those with an HDFs-angle > 70°.

Considering only the subgroup of patients having LVEF < 40%, the HDFs-angle maintained its association with the endpoint (HR 1.27 (95% CI 1.07–1.51), *p* = 0.006).

Clinical and echocardiographic characteristics were compared between patients having an HDFs-angle ≤ 70° and those having an HDFs-angle > 70° (Table 4). Patients with an HDFs-angle > 70° had a lower weight and generally better functional and morphological cardiac profile (higher LVEF, smaller LV EDVi, higher sphericity index, and higher LA reservoir strain). They also had higher median values for HDFs-ab and lower values for HDFs-ls.

## 4. Discussion

This is the first study evaluating echocardiography-derived HDFs in NIDCM patients. It showed that HDFs, specifically HDFs-angle, is significantly associated with MACE onset. A higher HDFs-angle was associated with worse prognosis and maintained its predictive value after individual adjustment for possible confounders such as age, LVEF, or GLS. An HDFs-angle > 70° was associated with more than double the risk for MACE, while a value lower than 70° provide a good negative predictive value.

The evaluation of prognosis of NIDCM patients, especially in the absence of symptoms, is challenging [2]. Many tests provide crucial parameters [21] but some of them require time-consuming, expensive, or invasive investigations, such as dobutamine-stress echocardiography, contrast-enhanced CMR, cardio-pulmonary test, or cardiac catheterization, inevitably limiting their availability in everyday clinical practice. This study explored a new parameter associated with clinical endpoints that can be obtained from standard TTE, with no need for additional acquisitions, and thus may have a role in future routinary clinical management of NIDCM patients.

HDFs have been known for a long time [22,23], but a framework for simple non-invasive assessment was introduced only recently [8].The software used in this study allows us to estimate the main features of the HDF vector. The calculation is performed by integrating data on LV deformation and the exchange of momentum across the mitral and aortic valves, without requiring the measurement of blood flow velocity. HDFs, or intraventricular pressure gradients, drive blood motion during both LV ejection and filling. They represent the ultimate result of the interaction between LV regional deformation and blood movement. Abnormal HDFs or their misalignment reflect the alteration of LV function, geometry, or blood flow, and lead to further heart remodeling [23]. The information on tissue–blood interaction brought by HDFs assessment may help reveal a sub-optimal cardiac function not evident in terms of tissue motion and can be complementary to the information provided by strain analysis [8,23]. In this cohort, HDFs yielded prognostic information while conventional TTE parameters such as LVEF and GLS did not. The relationship between strain and outcomes in NIDCM is complex and influenced by multiple factors, including disease etiology, individual patient characteristics, and comorbidities. Therefore, strain measurements alone may not reliably predict outcomes in NIDCM.

HDFs can be decomposed into apex-base (longitudinal) and lateral-septal (transversal) components, while HDFs-angle represents a combination of the forces acting in these two directions [9]. Indeed, the relative weight of these components determines the HDFs-angle, which ranges from 0° (fully transversal forces) to 90° (fully longitudinal forces). HDFs-ab was very low in the present cohort compared to values previously reported in healthy subjects, in which HDF values were more than double. Average HDFs-ab was 5.6% in our cohort compared to around 15% in healthy subjects [24,25], and average HDFs-ls was 1.6% in our cohort while a value of 2.4% was reported in healthy subjects [25]. HDFs-ab showed no significant role in MACE prediction in our cohort, neither over the whole cardiac cycle nor over the measured sub-phases. In this respect, it is worth noting that HDFs-ab showed a marked association (R > 0.5) with conventional indices of systolic function (LVEF and GLS), which did not show prognostic value in our cohort. We speculate that the moderate to severe systolic dysfunction observed in the present study may result in generally impaired HDFs-ab, irrespectively of further, future clinical worsening. Conversely, HDFs-angle showed lower correlation with LVEF and GLS, while HDFs-ls showed no correlations, therefore probably capturing a different and independent risk factor in this cohort.

In a recent study on HDF analysis by CMR in patients with dilated cardiomyopathy, impaired HDFs-ab, and the presence of HDFs-ab reversal during LV Suction were associated with worse myocardial mechanics and prognosis [16]. It must be noted that HDF absolute values reported in that study [16] were slightly higher than those observed in our cohort. Such differences may be due to the technique itself (CMR vs. TTE) but also to the different characteristics of the two populations. For instance, the patients enrolled in the present study were younger, had better functional class, and were not hospitalized.

**Figure 4:**
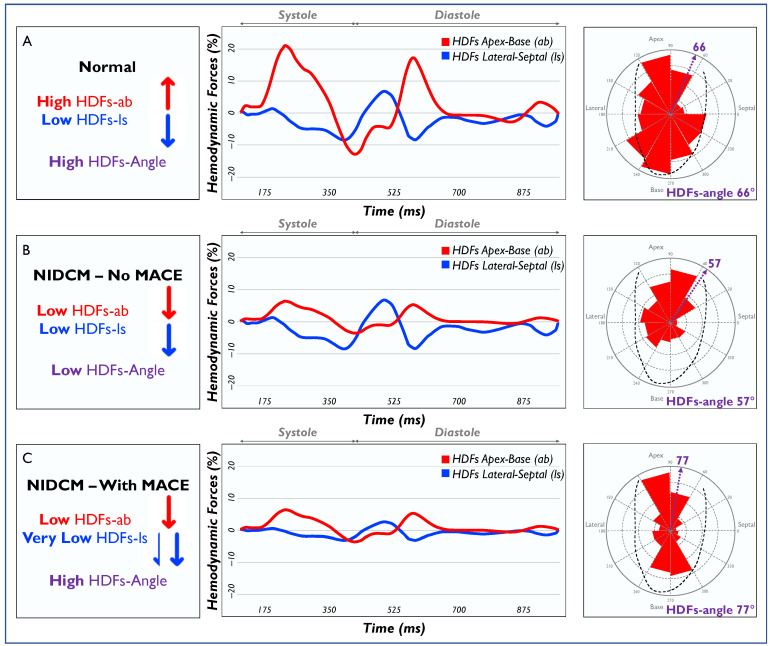
Examples of HDF components and orientation (angle) are displayed in healthy subjects (**A**) and NIDMC patients, with (**B**) and without (**C**) MACE. HDFs: hemodynamic forces; HDFs-ab: longitudinal HDFs; HDFs-ls: transversal HDFs; MACE: major adverse cardiovascular events; NIDCM: non-ischemic dilated cardiomyopathy.

Both low HDFs-ls and, even more, high HDFs-angle were associated with a worse prognosis in this cohort. This result may be in contrast with previous studies, which reported that high HDFs-ls forces were associated with a greater degree of desynchrony [11,15]. Relatively higher HDFs-ls and low or normal HDFs-ab result in lower HDFs-angle. However, in our cohort, patients with MACE had a high HDFs-angle, which is due to low HDFs-ab and even lower HDFs-ls relative to the HDFs-ab (Figure 4). This result could indicate that in NIDCM, a higher risk of cardiovascular complications may be due to a decrease in the transversal component of LV mechanics rather than to desynchrony itself. The importance of the transversal component of myocardial contraction is well known, as it counterbalances the reduction in longitudinal shortening in order to maintain systolic function [26,27,28]. In this context, HDFs-angle, representing both HDFs components, and being relatively independent from systolic function parameters and the time intervals of the cardiac cycle, may be a stronger predictor of MACE than longitudinal and transversal forces in isolation. A high HDFs-angle may mark a worsening myocardial performance occurring after LVEF impairment, capturing a reduction in transversal forces happening in patients with established loss of LV longitudinal function. To be noted, most of the patients with an HDFs-angle >70° had an LVEF higher than 35%. LVEF and other conventional TTE parameters failed to show a prognostic value in the total cohort and in this subgroup, while HDFs-angle was strongly associated with events in both. Therefore, the HDFs-angle may identify an independent risk factor, especially in patients with a moderate LV systolic dysfunction. If confirmed by further longitudinal studies, HDFs-angle may be used as a marker for future clinical events, in particular in asymptomatic patients with NIDCM with an uncertain prognosis.

In our cohort, conventional TTE parameters, such as LVEF and GLS, did not have prognostic value, which is in disagreement with previous studies [5,6,29]. We speculate that this dissimilarity may be due to differences in the characteristics of the cohort. In particular, the cohort included in this study was highly selected (with older age, and smaller LV), possibly reducing the prognostic value of these parameters.

If the prognostic value presented in this exploratory analysis is confirmed by prospective studies, HDFs analysis may become a key functional descriptor of the LV. Indeed, present results lead to a growing body of evidence highlighting the relevant associations of these mechanistic descriptors. For example, previous studies showed that HDFs have the ability to describe LV response to resynchronization therapy [11] and LV diastolic function [10]. Moreover, a study reported differences comparing patients with HF and normal or reduced EF and healthy volunteers, highlighting a potential for early detection of systolic dysfunction [12]. Thus, HDF analysis offers new insights on LV function, and it can easily be performed with standard TTE acquisitions, without the need for additional time-consuming investigations.

## 5. Limitations

This study has some limitations that must be considered. First, the study is single-center and retrospective and the sample size is relatively small, with limited rates of MACE. To limit possible confounders, patients with significant valvular heart disease, atrial fibrillation, low functional classes, and those hospitalized during TTE were excluded. Thus, present results must not be generalized to the whole NIDCM population or other cardiomyopathies. Further studies, possibly multicentric prospective with broader inclusion criteria, are certainly needed to confirm the findings of this analysis. Similarly, although HDFs analysis is feasible in patients with atrial fibrillation, an irregular cardiac cycle may represent a possible confounder.

A second limitation is that diastolic function information, in particular E/e′ ratio, was not available for all patients. Since many patients underwent first TTE evaluation before 2016, when the latest recommendations on the evaluation of LV diastolic function were published [20], tissue Doppler imaging was not routinely performed. However, the prognostic relevance of E/e′ and the added value of HDFs-angle were evident in the subset of patients having both parameters available.

Another limitation is the lack of an external validation cohort. However, the identification of cut-off values was not the aim of the present study, and the split values of HDFs identified must be intended as merely descriptive.

Finally, reproducibility of HDF measurement was not performed in the present study. However, excellent inter- and intra-observer reproducibility of TTE-derived HDF analysis was previously demonstrated [11,24,25,30].

## 6. Conclusions

Echocardiography-derived hemodynamic forces analysis has prognostic potential in asymptomatic patients with non-ischemic, non-valvular cardiomyopathy. In this cohort, HDFs-angle is associated with MACE onset while conventional echocardiographic parameters are not. The analysis of hemodynamic forces, which do not require additional acquisitions, might be included in the evaluation of patients with cardiomyopathies. HDFs-angle may be used as a marker of subclinical myocardial dysfunction and of risk of future clinical adverse events, especially in asymptomatic patients with NIDCM with uncertain prognosis.

## Figures and Tables

**Table 1:** Clinical, echocardiographic, and HDF characteristics of the study population.

	Total Population (*n*. 97)	No MACE (*n*. 78)	MACE (*n*. 19)	*p* Value
*Clinical variables*				
Age (years)	62 ± 14	59.4 ± 14.0	73.8 ± 9.4	**<0.001**
Male sex, *n* (%)	67 (69%)	59 (76%)	8 (42%)	0.071
Weight (kg)	75 ± 15	76 ± 15	71 ± 16	0.176
Height (cm)	168 ± 10	169 ± 10	161 ± 9	**0.002**
BSA (m^2^)	1.84 ± 0.21	1.86 ± 0.20	1.74 ± 0.21	**0.032**
BMI (kg/m^2^)	26.5 ± 4.5	26.5 ± 4.5	27.0 ± 5.0	0.728
Obesity, *n* (%)	19 (20%)	16 (21%)	3 (16%)	0.975
Hypertension, *n* (%)	50 (52%)	36 (46%)	14 (74%)	**0.031**
Dyslipidemia, *n* (%)	42 (43%)	31 (40%)	11 (58%)	0.152
Diabetes, *n* (%)	28 (29%)	20 (26%)	8 (42%)	**0.041**
COPD, *n* (%)	9 (9%)	7 (9%)	2 (11%)	0.834
CKD, *n* (%)	5 (5%)	3 (4%)	2 (11%)	0.260
Peripheral artery disease, *n* (%)	5 (5%)	3 (4%)	2 (11%)	0.260
Previous or active cancer, *n* (%)	13 (13%)	10 (13%)	4 (21%)	0.465
Previous heart failure hosp., *n* (%)	13 (13%)	11 (14%)	2 (11%)	0.682
Charlson Comorbidity Index	3 (1–4)	2 (1–3)	5 (3–6)	**<0.001**
Serum creatinine (mg/dL)	0.88 (0.75–1.05)	0.86 (0.74–1.05)	0.94 (0.82–1.12)	0.075
Hemoglobin (g/dL)	13.7 ± 1.9	14.0 ± 1.8	12.5 ± 1.6	**0.002**
Heart rate (bpm)	72 ± 16	73 ± 16	71 ± 14	0.612
QRS duration (ms)	117 ± 37	114 ± 36	129 ± 37	0.115
QRS ≥ 120 ms, *n* (%)	49 (51%)	35 (45%)	14 (74%)	**0.024**
Systolic BP (mmHg)	128 ± 17	128 ± 17	131 ± 18	0.514
Diastolic BP (mmHg)	75 ± 12	75 ± 12	74 ± 12	0.637
Follow-up time (years)	4.2(3.1–5.1)	4.2 (3.3–5.3)	4.1 (2.9–5.4)	0.620
*Medications, use of*				
Beta blockers, *n* (%)	52 (54%)	38 (49%)	14 (74%)	0.050
ACE-Inhibitors/ARBs, n (%)	68 (70%)	52 (67%)	16 (84%)	0.134
MRAs, *n* (%)	28 (29%)	19 (24%)	9 (47%)	**0.047**
Loop diuretics, *n* (%)	29 (30%)	19 (24%)	10 (53%)	**0.016**
Ivabradine, *n* (%)	13 (13%)	10 (13%)	3 (16%)	0.715
*Echocardiography*				
Interventricular septum (mm)	11.0 ± 1.9	10.9 ± 1.7	11.5 ± 2.5	0.356
Inferior-lateral wall (mm)	10.2 ± 1.9	10.0 ± 1.7	10.7 ± 2.3	0.270
RWT	0.37 ± 0.09	0.35 ± 0.08	0.41 ± 0.1	**0.042**
LV EDVi (mL/m^2^)	69 (60–82	68 (60–84)	70 (52–78)	0.676
LVMi (g/m^2^)	133 ± 32	134 ± 34	133 ± 35	0.870
Sphericity index	0.58 (0.53–0.64)	0.58 (0.54–0.64)	0.58 (0.53–0.67)	0.927
LVEF (%)	39.2 ± 8.6	39.1 ± 8.3	39.3 ± 10.2	0.948
LVEF < 40%, n (%)	47 (49%)	38 (49%)	9 (47%)	0.916
GLS (%)	−13.6 ± 3.6	−13.7 ± 3.6	−13.0 ± 3.9	0.514
GCS (%)	−17.1 ± 4.6	−16.9 ± 4.4	−17.9 ± 5.5	0.722
TAPSE (mm)	20 (18–23)	20 (18–22)	22 (20–25)	**0.037**
RV FWS (%)	−22.1 ± 4.4	−21.7 ± 4.6	−23.7 ± 3.7	0.094
LAVi (mL/m^2^)	30 (24–40)	29 (22–38)	34 (27–41]	0.269
LA reservoir strain (%)	21.2 ± 8.2	21.7 ± 8.2	19.0 ± 8.4	0.206
E wave (m/s)	0.63 (90.52–0.79)	0.61 (0.51–0.74)	0.67 (0.56–0.88)	0.137
E/A ratio	0.75 (0.64–1.09)	0.75 (0.64–1.09)	0.74 (0.58–1.05)	0.587
E/e’ ratio *	11.3 (7.5–16.1)	10.2 (7.3–14.2)	16.4 (11.6–20.3)	**0.011**
*Hemodynamic forces (HDFs)*				
HDFs apex-base (%)	5.6 (4.4–6.7)	5.7 (4.5–6.8)	5.0 (4.4–6.3)	0.375
HDFs lateral-septal (%)	1.6 (1.3–2.0)	1.7 (1.3–2.0)	1.4 (1.0–1.9)	**0.015**
HDFs-angle (°)	68 (64–71)	68 (63–71)	71 (67–75)	**0.005**
HDFs-angle > 70°, *n* (%)	30 (31%)	20 (26%)	10 (53%)	**0.022**
Systolic Thrust (%)	6.0 (4.5–7.6)	6.2 (4.6–7.6)	5.3 (4.5–7.0)	0.140
LV Suction (%)	−2.7 (−3.7;−2.0)	−2.6 (−3.7;−1.9)	−2.8 (−4.2;−2.1)	0.519
Diastolic Deceleration (%)	2.6 (1.8–3.9)	2.7 (1.8–3.9)	2.0 (1.8–4.0)	0.581
LV Suction Reversal, n (%)	52 (54%)	42 (54%)	10 (53%)	0.924
Systolic Thrust angle (°)	74 (70–78)	74 (69–78)	76 (72–78)	**0.016**
LV Suction angle (°)	59 (53–66)	58 (53–65)	63 (58–73)	**0.029**

* Data available for 54 patients (43 without and 11 with MACE).

**Table 2:** Univariate Cox regression analysis for MACE.

	Hazard Ratio [95% CI]	*p* Value
*Clinical variables*		
Age (per year)	1.09 (1.04–1.15)	**<0.001**
Male sex	0.40 (0.16–0.99)	**0.049**
Weight (per kg)	0.98 (0.94–1.01)	0.175
Height (per cm)	0.93 (0.88–0.97)	**0.002**
BSA (per m^2^)	0.07 (0.01–0.69)	**0.023**
BMI (per kg/m^2^)	1.03 (0.93–1.14)	0.598
Obesity	1.06 (0.35–3.20)	0.919
Hypertension	3.12 (1.12–8.68)	**0.030**
Dyslipidemia	1.96 (0.79–4.89)	0.147
Diabetes	2.65 (1.07–6.55)	**0.035**
COPD	1.37 (0.31–5.98)	0.675
CKD	1.96 (0.45–8.52)	0.368
Peripheral artery disease	2.39 (0.55–10.37)	0.246
Previous or active cancer	1.39 (0.80–2.43)	0.245
Previous heart failure hosp.	0.86 (0.20–3.71)	0.836
Charlson Comorbidity Index (per unit)	1.71 (1.34–2.18)	**<0.001**
Serum creatinine (per mg/dL)	1.20 (0.89–1.62)	0.240
Hemoglobin (per g/dL)	0.65 (0.49–0.85)	**0.002**
Heart rate (per bpm)	0.99 (0.97–1.03)	0.838
QRS duration (per ms)	1.01 (0.99–1.02)	0.200
QRS ≥ 120 ms	2.70 (0.97–7.57)	0.058
Systolic BP (per mmHg)	1.01 (0.99–1.04)	0.384
Diastolic BP (per mmHg)	0.99 (0.96–1.04)	0.924
*Medications, use of*		
Beta blockers	1.76 (0.95–3.27)	0.073
ACE-Inhibitors/ARBs	1.42 (0.78–2.59)	0.257
MRAs	1.44 (0.90–2.28)	0.127
Loop diuretics	2.75 (1.12–6.77)	**0.028**
Ivabradine	1.15 (0.34–3.96)	0.823
*Echocardiography*		
Interventricular septum (per mm)	1.15 (0.92–1.45)	0.223
Inferior-lateral wall (per mm)	1.14 (0.92–1.42)	0.247
RWT (per unit)	1.05 (1.01–1.09)	**0.019**
LV EDVi (per mL/m^2^)	1.00 (0.98–1.02)	0.948
LVMi (per g/m^2^)	1.00 (0.98–1.01)	0.739
Sphericity index (per unit)	1.03 (0.96–1.09)	0.442
LVEF (per %)	1.00 (0.95–1.06)	0.954
LVEF < 40%	1.14 (0.46–2.82)	0.785
GLS (per %)	1.01 (0.89–1.15)	0.909
GCS (per %)	0.97 (0.87–1.08)	0.589
TAPSE (per mm)	1.17 (1.02–1.34)	**0.025**
RV FWS (per %)	0.92 (0.81–1.04)	0.200
LAVi (per mL/m^2^)	1.01(0.97–1.05)	0.621
LA reservoir strain (per %)	0.95 (0.89–1.01)	0.097
E wave (per m/s)	2.36 (0.50–11.19)	0.281
E/A ratio (per unit)	1.04 (0.44–2.49)	0.926
E/e’ ratio (per unit) *	1.11 (1.02–1.20)	**0.012**
*Hemodynamic forces*		
HDFs Apex-Base (per %)	0.86 (0.66–1.13)	0.290
HDFs Lateral-Septal (per %)	0.27 (0.09–0.80)	**0.019**
HDFs-angle (per °)	1.16 (1.04–1.30)	**0.007**
HDFs-angle > 70°	2.68 (1.09–6.60)	**0.032**
Systolic Thrust (per %)	0.88 (0.73–1.07)	0.190
LV Suction (per %)	0.88 (0.62–1.26)	0.489
Diastolic Deceleration (per %)	0.96 (0.70–1.31)	0.774
LV Suction Reversal	1.08 (0.44–2.67)	0.869
Systolic Thrust angle (per °)	1.09 (0.99–1.20)	0.075
LV Suction angle (per °)	1.05 (1.00–1.11)	**0.041**

* Data available for 54 patients (11 events). Abbreviations as in Table 1.

**Table 3:** Bivariate Cox regression for individual adjustment of HDFs-angle.

	Hazard Ratio [95% CI]	*p* Value
Age (per year)	1.09 (1.03–1.14)	0.001
HDFs-angle (per °)	1.11 (1.00–1.23)	0.042
	*p* = 0.030 for change in −2Log Likelihood	
Charlson Comorbidity Index (per unit)	1.63 (1.29–2.06)	<0.001
HDFs-angle (per °)	1.13 (1.02–1.26)	0.021
	*p* = 0.013 for change in −2Log Likelihood	
Hemoglobin (per g/dL)	0.64 (0.49–0.84)	0.001
HDFs-angle (per °)	1.18 (1.06–1.33)	0.004
	*p* = 0.001 for change in −2Log Likelihood	
LVEF (per %)	0.96 (0.90–1.02)	0.188
HDFs-angle (per °)	1.20 (1.06–1.36)	0.003
	*p* = 0.001 for change in −2Log Likelihood	
LV EDVi (per mL/m^2^)	1.01 (0.99–1.03)	0.325
HDFs-angle (per °)	1.19 (1.05–1.34)	0.005
	*p* = 0.002 for change in −2Log Likelihood	
GLS (per %)	1.12 (0.96–1.29)	0.142
HDFs-angle (per °)	1.20 (1.07–1.35)	0.002
	*p* = 0.001 for change in −2Log Likelihood	
TAPSE (per mm)	1.19 (1.03–1.37)	0.019
HDFs-angle (per °)	1.17 (1.05–1.30)	0.006
	*p* = 0.003 for change in −2Log Likelihood	
LA reservoir strain (per %)	0.94 (0.88–0.99)	0.046
HDFs-angle (per °)	1.17 (1.05–1.30)	0.003
	*p* = 0.002 for change in −2Log Likelihood	
E/e′ ratio (per unit) *	1.12 (1.02–1.23)	0.017
HDFs-angle (per °)	1.18 (1.02–1.37)	0.028
	*p* = 0.013 for change in −2Log Likelihood	

* Data available for 54 patients (11 events). Abbreviations as in Table 1.

**Table 4:** Comparison of clinical, echocardiographic, and HDFs characteristics according to the HDFs-angle value.

	HDFs-Angle ≤ 70° (n. 67)	HDFs-Angle > 70° (n. 30)	*p* Value
*Clinical variables*			
Age (years)	60.9 ± 14.4	65.1 ± 14.3	0.195
Male sex, n (%)	50 (75%)	17 (57%)	0.077
Weight (kg)	77 ± 16	70 ± 13	**0.040**
Height (cm)	169 ± 9	166 ± 12	0.288
BSA (m^2^)	1.86 ± 0.21	1.78 ± 0.21	0.066
BMI (kg/m^2^)	27.0 ± 4.8	25.6 ± 4.0	0.139
Obesity, n (%)	17 (25%)	4 (13%)	0.286
Hypertension, n (%)	33 (49%)	17 (57%)	0.500
Dyslipidemia, n (%)	28 (42%)	14 (47%)	0.654
Diabetes, n (%)	20 (30%)	8 (27%)	0.749
COPD, n (%)	7 (10%)	2 (7%)	0.716
CKD, n (%)	4 (6%)	1 (3%)	0.567
Peripheral artery disease, n (%)	3 (5%)	2 (7%)	0.643
Previous or active cancer, n (%)	9 (13%)	5 (17%)	0.664
Previous heart failure hosp., n (%)	11 (14%)	2 (11%)	0.682
Charlson Comorbidity Index	3 (1–4)	3 (1–4)	0.865
Serum creatinine (mg/dL)	0.88 (0.75–1.05)	0.91 (0.77–1.05)	0.917
Hemoglobin (g/dL)	13.7 ± 1.7	13.7 ± 2.1	0.953
Heart rate (bpm)	72 ± 17	72 ± 14	0.884
QRS duration (ms)	117 ± 36	117 ± 38	0.981
QRS ≥ 120 ms, n (%)	34 (51%)	15 (40%)	0.946
Systolic BP (mmHg)	128 ± 17	129 ± 16	0.854
Diastolic BP (mmHg)	75 ± 12	74 ± 12	0.719
Follow-up time (years)	4.2 (3.2–5.1)	3.4 (3.0–5.4)	0.421
*Medications, use of*			
Beta blockers, n (%)	34 (56%)	18 (64%)	0.447
ACE-Inhibitors/ARBs, n (%)	47 (71%)	21 (72%)	0.905
MRAs, n (%)	19 (29%)	9 (30%)	0.939
Loop diuretics, n (%)	18 (27%)	11 (37%)	0.330
Ivabradine, n (%)	9 (13%)	4 (16%)	0.989
*Echocardiography*			
Interventricular septum (mm)	10.9 ± 1.9	11.2 ± 2.0	0.599
Inferior-lateral wall (mm)	10.3 ± 2.0	9.9 ± 1.6	0.262
RWT	0.36 ± 0.10	0.38 ± 0.08	0.293
LV EDVi (mL/m^2^)	73 (64–97)	60 (53–70)	**<0.001**
LVMi (g/m^2^)	140 ± 36	121 ± 26	**<0.001**
Sphericity index	0.61 (0.55–0.67)	0.55 (0.52–0.59)	**<0.001**
LVEF (%)	36.6 ± 8.5	44.8 ± 5.6	**<0.001**
LVEF < 40%, n (%)	42 (63%)	5 (17%)	**<0.001**
GLS (%)	−12.6 ± 3.4	−15.8 ± 3.0	**<0.001**
GCS (%)	−16.9 ± 4.4	−17.9 ± 5.5	0.722
TAPSE (mm)	20 (18–23)	20 (19–22)	0.548
RV FWS (%)	−21.5 ± 3.5	−24.2 ± 3.6	**0.021**
LAVi (mL/m^2^)	31 (24–42)	28 (20–34)	0.064
LA reservoir strain (%)	19 (14–24)	24 (18–29)	**0.045**
E wave (m/s)	0.63 (0.53–0.82)	0.60 (0.49–0.76)	0.198
E/A ratio	0.75 (0.64–1.13)	0.76 (0.65–1.02)	0.958
E/e’ ratio^*^	11.1 (7.3–14.2)	11.6 (7.8–19.0)	0.524
*Hemodynamic forces (HDFs)*			
HDFs apex-base (%)	5.4 (4.2–6.2)	6.6 (4.9–8.1)	**0.001**
HDFs lateral-septal (%)	1.7 (1.3–2.1)	1.3 (1.0–1.7)	**0.001**
HDFs-angle (°)	66 (62–69)	73 (71–75)	**0.005**
Systolic Thrust (%)	5.6 (3.7–7.2)	6.9 (5.2–8.7)	**0.007**
LV Suction (%)	−2.5 (−3.2;−1.8)	−3.5 (−4.7;−2.8)	**<0.001**
Diastolic Deceleration (%)	2.3 (1.7–3.6)	2.9 (2.0–4.5)	0.051
LV Suction Reversal, n. (%)	34 (51%)	18 (60%)	0.398
Systolic Thrust angle (°)	72 (68–75)	78 (76–80)	**<0.001**
LV Suction angle (°)	57 (51–61)	67 (62–74)	**<0.001**

* Data available for 54 patients (11 events). Abbreviations as in Table 1.

## Data Availability

The data underlying this article will be shared on reasonable request to the corresponding author.

## Supplementary Materials

The following supporting information can be downloaded at: https://www.mdpi.com/article/10.3390/jcm13133862/s1. Table S1. Correlation between Hemodynamic forces and clinical and echocardiographic variables; Table S2. Mann-Whitney test for hemodynamic forces according to dichotomous variables; Figure S1. Box plots of hemodynamic forces angle by various categorical variables; Table S3. Bivariate Cox regression for individual adjustment of HDFs-angle, including variables potentially associated with the endpoint (*p* ≤ 0.200) on univariate analysis (variables not displayed in the main text); Figure S2. Receiver operating characteristics curve of MACE prediction by HDFsangle > 70°.
