# Supplementary material for: Echocardiography-Derived Hemodynamic Forces Are Associated with Clinical Outcomes in Patients with Non-Ischemic Dilated Cardiomyopathy
Source: J Clin Med. 2024 Jun 30;13(13):3862. doi: 10.3390/jcm13133862 (PMC11242817; doi:10.3390/jcm13133862)

Supplementary Table S1.

Correlation between Hemodynamic forces and clinical and echocardiographic variables.

	**HDFs-ab**		**HDFs-ls**		**HDFs-angle**	
	**R**	**p value**	**R**	**p value**	**R**	**p value**
Clinical variables						
**Age (per year)**	**-0.302**	**0.003**	**-0.290**	**0.004**	0.151	0.140
**Weight (per kg)**	-0.124	0.226	0.008	0.938	-0.144	0.159
**Height (per cm)**	0.108	0.293	0.175	0.087	-0.187	0.066
**BSA (per m^2)**	-0.055	0.591	0.066	0.522	-0.176	0.085
**BMI (per kg/m^2)**	-0.192	0.060	-0.103	0.318	-0.032	0.756
**Serum creatinine (per mg/dl)**	0.030	0.772	-0.035	0.735	0.070	0.502
**Hemoglobin (per g/dl)**	0.082	0.424	0.025	0.806	0.066	0.522
**Heart rate (per bpm)**	-0.026	0.798	0.083	0.420	-0.005	0.962
**QRS duration (per ms)**	**-0.201**	**0.049**	**-0.203**	**0.047**	0.037	0.717
**Systolic BP (per mmHg)**	0.065	0.527	0.095	0.357	0.026	0.803
**Diastolic BP (per mmHg)**	-0.144	0.160	-0.019	0.855	-0.037	0.721
Echocardiography						
**Interventricular septum (per mm)**	0.091	0.377	0.073	0.480	0.070	0.498
**Inferior-lateral wall (per mm)**	0.061	0.555	0.136	0.185	0.021	0.838
**LV EDD (per mm)**	**-0.358**	**<0.001**	-0.165	0.106	**-0.287**	**0.004**
**RWT (per unit)**	**0.223**	**0.028**	0.177	0.082	0.165	0.105
**LV EDVi (per ml/m^2)**	-0.188	0.065	-0.013	0.897	**-0.279**	**0.006**
**LVMi (per g/m^2)**	**-0.204**	**0.045**	-0.049	0.633	-0.145	0.156
**Sphericity index (per unit)**	**-0.283**	**0.006**	-0.062	0.555	**-0.383**	**<0.001**
**LVEF (per %)**	**0.629**	**<0.001**	0.149	0.146	**0.438**	**<0.001**
**GLS (per %)**	**-0.577**	**<0.001**	-0.063	0.543	**-0.460**	**<0.001**
**GCS (per %)**	**-0.609**	**<0.001**	-0.170	0.096	**-0.405**	**<0.001**
**TAPSE (per mm)**	-0.089	0.385	-0.064	0.534	0.032	0.755
**RV FWS (per %)**	-0.241	0.055	0.060	0.636	-0.205	0.105
**LAVi (per ml/m^2)**	-0.154	0.132	-0.004	0.970	**-0.232**	**0.022**
**LA reservoir strain (per %)**	**0.208**	**0.040**	-0.007	0.948	0.172	0.093
**E wave (per m/s)**	-0.117	0.255	0.026	0.798	-0.123	0.231
**E/A ratio (per unit)**	0.015	0.891	-0.061	0.571	0.057	0.598
**E/e' ratio (per unit)***	-0.203	0.142	-0.235	0.087	0.120	0.387

BMI: body mass index; BP: blood pressure; BSA: body surface area; E wave: early filling wave at mitral inflow Doppler imaging; E/A ratio: ratio between E wave and atrial filling wave at mitral Doppler imaging; E/e' ratio: ratio between E wave and average septal and lateral early wave at tissue Doppler imaging (e'); GCS: global circumferential strain; GLS: global longitudinal strain; LA: left atrium; LAVi: LA volume indexed for BSA; LV EDVi: LV end-diastolic volume indexed for BSA; LVEF: LV ejection fraction; LVMi: left ventricular mass indexed for BSA; TAPSE: tricuspid annular plane systolic excursion; RV FWS: right ventricle free wall strain; RWT: relative wall thickness.

Supplementary Table S2.

Mann-Whitney test for hemodynamic forces according to dichotomous variables.

	**HDFs-ab**	**HDFs-ls**	**HDFs-angle**
	**p value**	**p value**	**p value**
Clinical variables			
**Male sex**	0.558	0.177	0.100
**Obesity**	0.358	0.868	0.311
**Hypertension**	0.154	0.350	0.945
**Dyslipidemia**	0.491	0.413	0.628
**Diabetes**	0.297	0.561	0.307
**COPD**	0.765	0.650	0.505
**CKD**	0.463	0.796	0.854
**Peripheral artery disease**	0.750	0.098	0.677
**Previous or active cancer**	**0.003**	**0.003**	0.524
**Previous heart failure hosp.**	0.175	0.452	0.570
**Charlson Comorbidity Index***	**0.050**	0.180	0.610
**QRS ≥ 120 ms**	0.114	0.230	0.781
Medications, use of			
**Beta blockers**	0.963	**0.009**	**0.039**
**ACE-Inhibitors/ARBs**	**0.047**	**0.004**	0.164
**MRAs**	0.227	**0.009**	0.144
**Loop diuretics**	0.332	**0.006**	**0.037**
**Ivabradine**	0.937	0.341	0.844
Echocardiography			
**LVEF < 40%**	**<0.001**	0.444	**<0.001**

*Kruskal-Wallis ANOVA for ordinal variable.

ACE: angiotensin-converting enzyme; ARBs: angiotensin II-receptor blockers; CKD: chronic kidney disease; COPD: chronic obstructive pulmonary disease; MRAs: mineralocorticoid receptor antagonists; LVEF: left ventricular ejection fraction.

Supplementary Figure S1. Box plots of hemodynamic forces angle by various categorical variables.

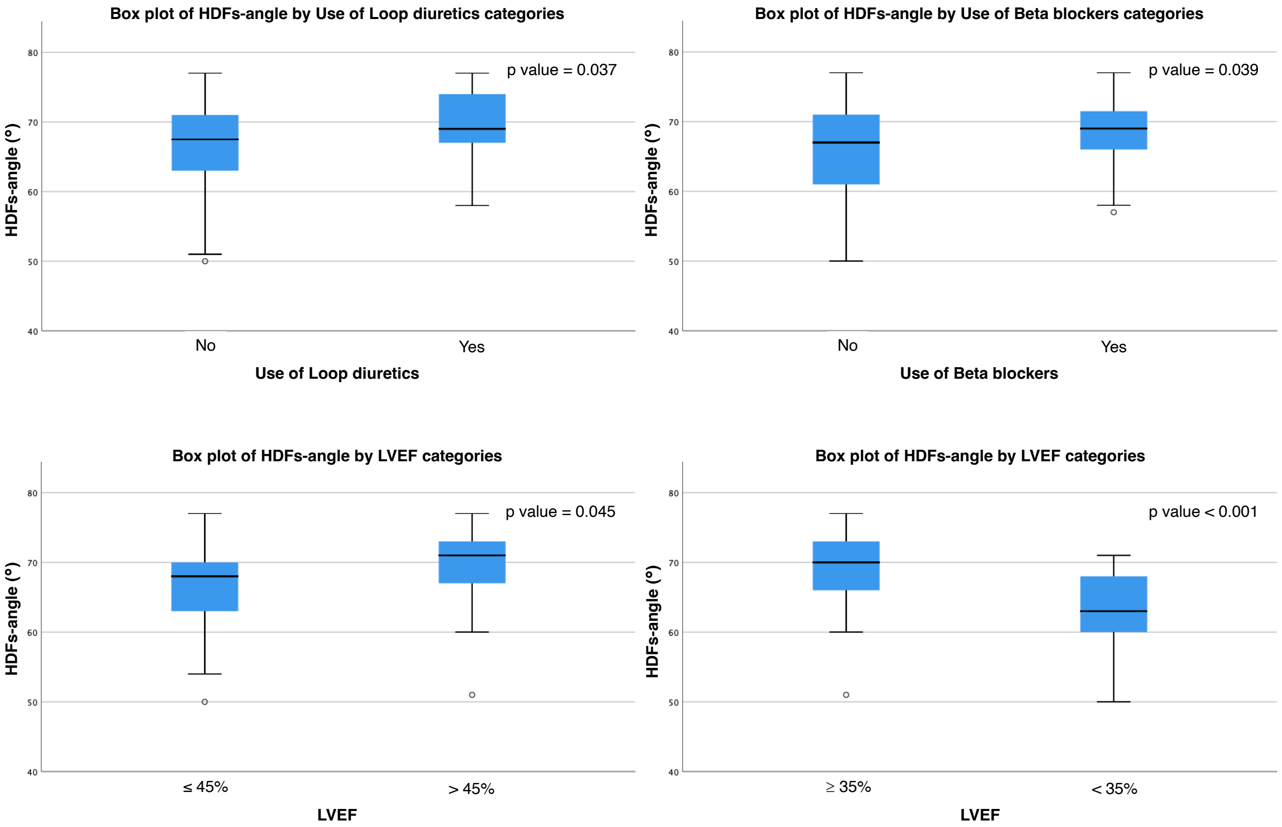

HDFs: hemodynamic forces; LVEF: left ventricular ejection fraction.

Supplementary Table S3.

Bivariate Cox regression for individual adjustment of HDFs-angle, including variables potentially associated with the endpoint (p≤0.200) on univariate analysis (variables not displayed in the main text).

	Hazard Ratio [95% CI]	p value
Clinical variables		
**Male sex**	0.46 [0.19-1.13]	0.091
**HDFs-angle (per °)**	1.16 [1.04-1.30]	0.011
**Weight (per kg)**	0.98 [0.94-1.02]	0.228
**HDFs-angle (per °)**	1.16 [1.04-1.29]	0.009
**Height (per cm)**	0.93 [0.89-0.98]	0.008
**HDFs-angle (per °)**	1.15 [1.02-1.29]	0.018
**BSA (per m^2)**	0.07 [0.01-0.85]	0.037
**HDFs-angle (per °)**	1.16 [1.04-1.30]	0.010
**Hypertension**	3.03 [1.08-8.47]	0.035
**HDFs-angle (per °)**	1.15 [1.04-1.27]	0.008
**Dyslipidemia**	2.08[0.83-5.21]	0.118
**HDFs-angle (per °)**	1.17 [1.05-1.32]	0.007
**Diabetes**	3.05 [1.23-7.55]	0.016
**HDFs-angle (per °)**	1.17 [1.05-1.31]	0.008
**QRS ≥ 120**	2.57 [0.92-7.22]	0.073
**HDFs-angle (per °)**	1.16 [1.04-1.29]	0.008
Medications		
**Use of Beta blockers**	1.73 [0.92-3.25]	0.087
**HDFs-angle (per °)**	1.14 [1.01-1.29]	0.038
**Use of MRAs**	1.41 [0.88-2.25]	0.102
**HDFs-angle (per °)**	1.17 [1.04-1.31]	0.009
**Use of Loop diuretics**	2.15 [0.86-5.34]	0.102
**HDFs-angle (per °)**	1.43 [1.03-1.28]	0.010
Echocardiography		
**RWT (per unit)**	1.05 [1.00-1.09]	0.033
**HDFs-angle (per °)**	1.16 [1.04-1.30]	0.010
**RV FWS (per %)**	0.95 [0.83-1.08]	0.418
**HDFs-angle (per °)**	1.17 [1.02-1.33]	0.010

Abbreviations as in Table S1 and S2.

Supplementary Figure S2. Receiver operating characteristics curve of MACE prediction by HDFs-angle >70°.

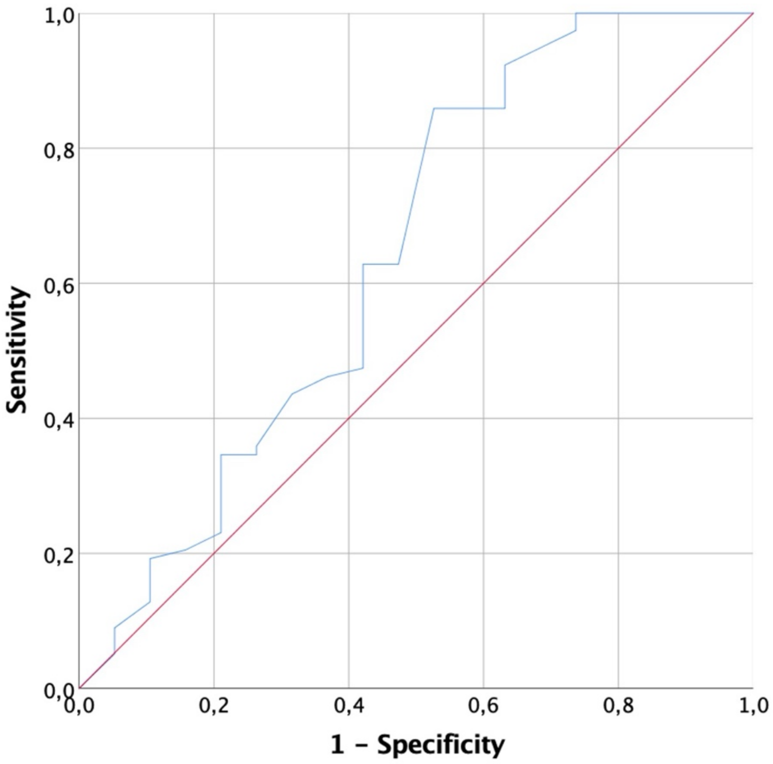

Supplement: Supplementary file 1 [file jcm-13-03862-s001.zip › jcm-3049229-supplementary.pdf]
